# Abdominal Pain and Examination of Hernial Orifices: The Forgotten Art of Physical Diagnosis

**DOI:** 10.7759/cureus.17486

**Published:** 2021-08-27

**Authors:** Sotirios G Doukas, Panagiotis G Doukas, Nagasri Upadrasta, Nayan Kothari

**Affiliations:** 1 Department of Medicine, Saint Peter's University Hospital, New Brunswick, USA; 2 Department of Surgery, Yale School of Medicine, New Haven, USA; 3 Department of Medicine, St. George's University School of Medicine, True Blue, GRD

**Keywords:** hernial orifice examination, hernia, clinical medicine, physical examination, physical diagnosis

## Abstract

Introduction: Acute abdominal pain can be the first manifestation of a hernial pathology. The estimated risk of incarcerated hernia is 1%-3% over a person's lifetime. Therefore, hernial orifice examination should be conducted routinely, especially in cases of abdominal pain. We hypothesized that physical examination of hernial orifices is not routinely performed and documented in patients presenting with acute abdominal pain.

Methods: A retrospective chart review of 100 patients who were evaluated for abdominal pain over a three-month time frame at our institution.

Results: From the 100 reviewed cases, the hernial orifice examination was performed in two cases by an Internal Medicine or Emergency Medicine physician (2%). Out of the eight cases with General Surgery consultation, only one case had hernial orifices examination (12.5%). In the 10 cases with Gastroenterology consultation, not a single case had hernial orifice examination.

Conclusion: We demonstrate that hernial examination is infrequently performed in clinical practice and suggest that emphasis should be placed on the efficient performance of physical examination and maintain the art of physical diagnosis.

## Introduction

The etiology of acute abdominal pain can vary in severity. Abdominal pain can be the first manifestation of the acute abdomen, which requires aggressive management, including surgical interventions to prevent devastating consequences. A well-performed history and physical examination may promote early diagnosis and proper management [1-4]. A hypothesis-driven physical examination is a cost-effective way to rule in and out medical conditions directing the following diagnostic and therapeutic steps appropriately [5].

The primary approach in acute abdominal pain is to narrow the differential diagnosis using history and physical examination and determine the best next step. A thorough physical examination also helps with avoiding unnecessary paraclinical testing and thereby delays in diagnosis and management. There are four components to an abdominal physical examination (inspection, percussion, palpation, and auscultation) along with some specialized maneuvers which are highly predictive of certain diseases. Something as basic as a visual inspection can provide a wealth of information. A complete physical examination in patients with abdominal pain requires examination of hernial orifices [1-4].

Hernia as a medical condition has been mentioned in history as early as the fifth century. Hippocrates referred to this pathology as "etru rhexis," which means rupture of the abdominal wall [6]. The prevalence of abdominal hernias is 1.7% for all ages and 4% for inpatients over 45 years of age. Inguinal hernias account for 75% of abdominal wall hernias, with a lifetime risk of 27% in men and 3% in women [7], and those protruding through the umbilical ring are umbilical hernias, accounting for 6% in adults (including paraumbilical hernia), mostly seen in middle-aged obese women, with a male to female ratio of about 1:3 [8]. Femoral and umbilical hernias occur more often in women. Incarcerated or strangulated hernia is a life-threatening complication with an estimated 1%-3% risk in a lifetime [9]. Emergent hernia repairs involving incarcerated or strangulated bowel is associated with increased morbidity and mortality. However, with properly timed medical management and elective surgical repair, hernia complications are avoidable and have favorable outcomes. Therefore, a hernial orifice examination should be conducted as part of a routine abdominal examination, especially in cases of abdominal pain.

We hypothesized that physical examination of hernial orifices is not routinely performed and documented in patients presenting with acute abdominal pain.

## Materials and methods

This study entails a retrospective chart review of 100 patients presented with abdominal pain at Saint Peter's University Hospital, New Brunswick, NJ, USA, in the period February 24, 2020 to May 31, 2020. The physical examination section of the ED, admission history and physical, and consultant notes were reviewed. The study's protocol was approved by the Committee for the Protection of Human Subjects in Research at Saint Peter's University Hospital. (Protocol Title: Examination of the Hernial Orifices in Patients with Acute Abdominal Pain - A Retrospective Chart Review: Study Protocol 20:34.)

For the sample selection, specific inclusion and exclusion criteria were used. We included patients who presented with abdominal pain as chief complaint during the selected period. Specifically, inpatients, patients under observation, and patients evaluated and discharged in the Emergency Department (ED patients) were included. In addition, documentation of physical examination performed by emergency physicians, Internal Medicine residents/attendings, gastroenterology fellows/attendings, and surgery residents/attendings was reviewed. The documentation was examined carefully for evidence of hernial orifice examination. Specifically, we searched for evidence of ventral, groin hernias, or pelvic hernial examination. We excluded patients <18 years old, patients without abdominal pain as chief complaint, patients presenting for elective outpatient procedures, and those admitted by the study's authors.

## Results

**Figure 1:**
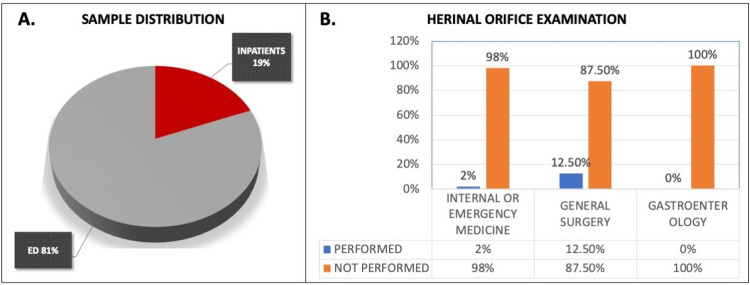
Documented cases of hernial orifices examination in patients with acute abdominal pain as a chief complaint. (A) The parentage of Inpatients and Emergency Department (ED) patients included (in absolute numbers: 81 ED patients and 19 inpatients). (B) The percentage of hernial orifices examinations performed and documented by Internal Medicine and Emergency Medicine, General Surgery, and Gastroenterology.

One hundred patient cases were included in this study. Of the 100 patients, 19 patients were admitted to the medical ward (inpatients) and 81 patients were treated in the ED without being admitted (ED visits). Of the 100 patients, 39 were males and 61 were females. The average age of the patients was 44 years old (Figure 1A).

From the 100 cases, the hernial orifice examination was documented to have been performed for two cases (2%) (Figure 1B). In one case, the hernial orifice examination was documented to have been performed by an ED physician and in the other case by an internal medicine resident. The General Surgery service was consulted in eight cases, and examination of hernial orifices was documented to have been performed in only one of the cases (12.5%). The General Surgeon and the ED physician documented a hernial orifice exam for the same case (Figure 1B). The Gastroenterology service was consulted in 10 cases, and examination of hernial orifices was not documented to have been performed in any of the cases (0%) (Figure 1B).

**Figure 2:**
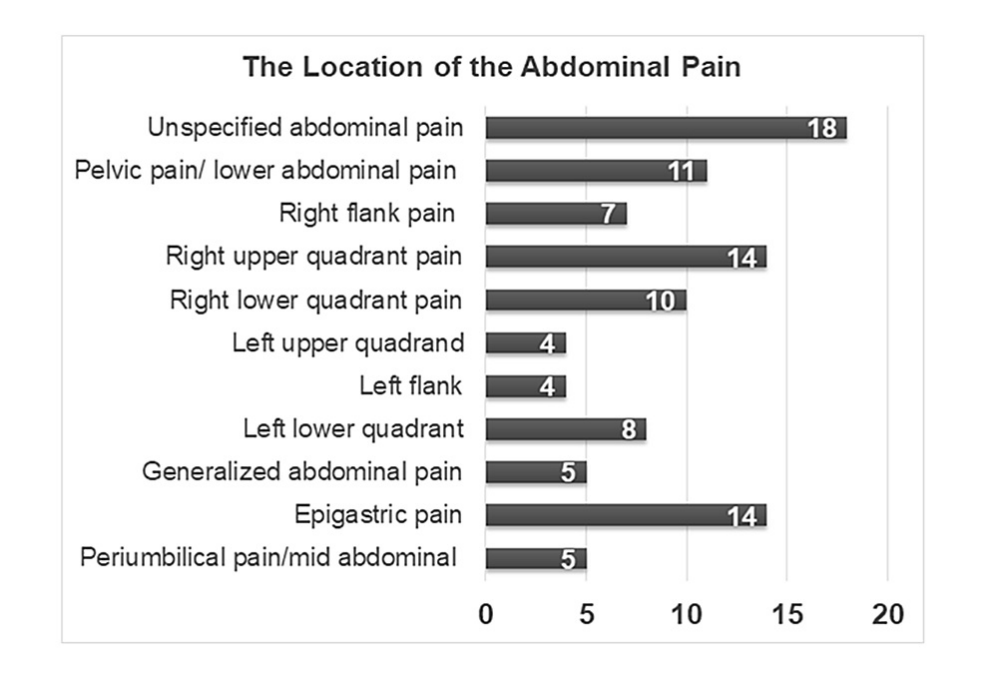
The location of abdominal pain at presentation: most of the patients had unspecific, right upper quadrant, or epigastric.

Followingly, the duration and location of the pain were estimated in all cases. Seventy patients presented with abdominal pain lasting less than four days, and 30 of them with abdominal pain lasting for weeks. None of the patients had abdominal pain for more than four weeks. The location of the pain varied, as depicted in Figure 2.

## Discussion

Abdominal pain is a common complaint, with approximately 5%-10% of the estimated ED visits be due to abdominal pain [10]. Acute abdominal pain usually has an onset of hours or days. On the other hand, a pain to be considered chronic should last or recur for more than three months [11]. The differential for abdominal pain is exhaustive. A careful evaluation with proper history and physical examination is necessary. The initial approach of the examiner should be to identify signs and symptoms that might indicate life-threatening conditions requiring prompt surgical and medical management. As discussed before, the literature suggests the performance of hernial orifice examination in every patient presenting with abdominal pain. Retrospective chart review showed that examination of hernial orifices is rarely performed in cases of acute abdominal pain. Specifically, our data showed that hernial orifice examination was documented to have been performed only in 2% of the cases by internal medicine residents and physicians and in 12.5% (1/8) by general surgery residents and attendings. Given that the lifetime risk for inguinal hernias is 27% in men and 3% in women, a hernial examination is of high importance, and it should be performed and documented in every patient complaining of acute abdominal pain [7].

Inguinal hernias are the most common type of hernias and a proper physical examination has a sensitivity of 74.5% and specificity of 96.3% for their detection [12]. The examination starts with inspecting the inguinal area for bulges or asymmetry, followed by palpation of the scrotum and towards the external inguinal ring if the patient is a male. Finally, the inguinal canal should be carefully examined. Although the examination should optimally be performed with the patient standing, it can be performed with the patient in a supine position if the patient cannot do so. The patients should be prompted to cough or bear down or perform a Valsalva maneuver to increase the intra-abdominal pressure and reveal the hernia [1,13]. Both right and left inguinal areas should be examined and compared throughout the examination. Auscultation of detected inguinal hernias is recommended since bowel sounds might indicate intestinal herniation. If a hernia is detected, the examiner should assess if the hernia is reducible or irreducible [1]. For a complete assessment of hernial orifices, the examiner should subsequently perform inspection and palpation of the epigastric, direct inguinal, indirect inguinal, incisional, umbilical, and femoral hernial orifices for signs of herniation [1].

Although most of the hernias are often asymptomatic, various clinical manifestations can be commonly recognizable. Some common symptoms for hernial strangulation include a painless bulge in the hernia site, and abdominal pain followed by nausea and vomiting [1]. During the physical examination, the bulginess in the hernial site commonly appears erythematous. Worsening of the pain in combination with constitutional signs suggesting sepsis, such as fever, tachycardia, and hypotension, might indicate strangulation [1]. Progression of strangulation to the devastating event of peritonitis leads to a more toxic appearance, generalized abdominal pain, shock, or even death [1]. Progression of symptoms to involuntary guarding, rebound tenderness, and cessation of bowel sounds, suggest peritonitis and prompt surgical intervention is suggested [1]. In our study, many patients had either right or left lower quadrant, pelvic, or generalized abdominal pain. In these patients, although the history could suggest an alternative diagnosis, hernial orifice examination is indicated, given the location of the pain.

During a clinical encounter, the physician should act as an investigator and collect clues that will lead to the diagnosis of the disease. Although science provided a tremendous number of imaging and laboratory investigations to diagnose most diseases, the effectiveness of a properly executed history and physical exam is enough to determine most of the common medical conditions [14]. However, despite these facts, recent studies suggest that nowadays, both attendings and residents spend limited time with the patient and instead devote more of their time for documentation and data collection through the electronic medical record system [15]. This significant decrease in the physician's time with the patients can potentially lead to atrophy of the previously gained clinical skills and compromise the physician's overall clinical capacity.

In contrast to the common belief, technological evolution continuously provides ever-increasing opportunities to diagnose medical conditions at the bedside. Ultrasound remains the best initial diagnostic modality to diagnosed inguinal hernias in patients with equivocal physical findings [16]. Multiple protocols and clinical guides have been developed to use point-of-care ultrasound for the accurate assessment of the abdomen and hernial orifices [17].

The current reality of a devastating pandemic could negatively affect the physical diagnosis, with the addition of necessary regulations that decrease the physical contact between the clinician and the patient [18,19]. Although it is too early to see what exact effect the COVID-19 pandemic had on medical students, residents, and attending physicians, clues presage its negative impact. National evaluation examinations such as USMLE Step 2 CS have been urgently discontinued to minimize exposure to the new coronavirus [20]. Also, clinical rotations have been modified to adjust to the new reality [18,19]. We strongly believe that maintenance to improve and maintain a high quality of care, the educational and healthcare system should develop novel and evidence-based methods to teach clinical skills.

Although this study provides evidence that hernial orifice examination is not commonly performed and documented in patients presenting with acute abdominal pain, several limitations applied: (1) This is a retrospective study of a single community medical institution, and therefore studies including other institutions should be performed. (2) The study relies on clinical documentation to determine whether an examination was performed. Ideally, clinical documentation should represent the physical examination maneuvers performed. However, it is possible that a hernial orifice examination was actually performed but not documented in some of the cases included in this study.

## Conclusions

Overall, our study provides evidence that examining the hernial orifices is infrequently performed and documented in clinical practice at our institution. We suggest that Emergency Medicine physicians, Internal Medicine physicians, and Specialists should perform and document the examination of the hernial orifices in every patient encounter with a chief complaint of abdominal pain, as the literature suggests. We support the power of clinical skills and physical diagnosis as a way to maintain a cost-effective, evidence-based, and patient-centered clinical practice. Finally, we suggest that emphasis should be placed on the efficient performance of physical examination and maintain the art of physical diagnosis.
